# Distinct gene expression profiles between primary breast cancers and brain metastases from pair-matched samples

**DOI:** 10.1038/s41598-019-50099-y

**Published:** 2019-09-16

**Authors:** Takayuki Iwamoto, Naoki Niikura, Rin Ogiya, Hiroyuki Yasojima, Ken-ichi Watanabe, Chizuko Kanbayashi, Michiko Tsuneizumi, Akira Matsui, Tomomi Fujisawa, Tsutomu Iwasa, Tadahiko Shien, Shigehira Saji, Norikazu Masuda, Hiroji Iwata

**Affiliations:** 1 Departments of Breast and Endocrine Surgery, Okayama University Hospital, , Okayama, Japan; 2 Department of Breast and Endocrine Surgery, Tokai University School of Medicine, Isehara, Japan; 3 Department of Breast surgery, Osaka National Hospital, Osaka, Japan; 4 Department of Breast Surgery, Hokkaido Cancer Center, Sapporo, Japan; 5 Department of Breast Oncology, Niigata Cancer Center Hospital, Niigata, Japan; 6 Department of Breast surgery, Shizuoka General Hospital, Shizuoka, Japan; 7 Department of Surgery, National Hospital Organization, Tokyo Medical Center, Tokyo, Japan; 8 Department of breast oncology, Gunma Prefectural Cancer Center, Ohta, Japan; 9 Department of Medical Oncology, Kinki University School of Medicine, Osaka-Sayama, Osaka, Japan; 10 Department of Medical Oncology, Fukushima medical university, Fukushima, Japan; 11 Department of Breast Oncology, Aichi Cancer Center Hospital, Nagoya, Japan

**Keywords:** Breast cancer, Microarrays

## Abstract

Our objectives were to determine whether clinic-pathological markers and immune-related gene signatures in breast cancer exhibit any change upon brain metastasis and whether previously reported genes significantly associated with brain metastases and the epithelial-mesenchymal transition (EMT) were reproducible and consistent in our dataset. Sixteen pair-matched samples from primary breast cancers and brain metastases diagnosed were collected from the Japan Clinical Oncology Group Breast Cancer Study Group. Gene expression profiles for immune-, brain metastases-, and EMT-related genes were compared between primary breast cancers and brain metastases. Potential therapeutic target genes of 41 FDA-approved or under-investigation agents for brain metastases were explored. Immune-related signatures exhibited significantly lower gene expression in brain metastases than in primary breast cancers. No significant differences were detected for the majority of genes associated with brain metastases and EMT in the two groups. Among 41 therapeutic target candidates, *VEGFA* and *DNMT3A* demonstrated significantly higher gene expression in brain metastases. We found that distinct patterns of gene expression exist between primary breast cancers and brain metastases. Further studies are needed to explore whether these distinct expression profiles derive from or underlie disease status and compare these features between metastases to the brain and other sites.

## Introduction

The incidence of brain metastases in patients with cancer is rising, likely because many patients survive longer owing to the improvement of systemic therapies to control extracranial disease; thus, patients can experience brain metastases who previously may have died sooner from other disease manifestations Brain metastases constitute devastating complications of cancer for which there is no effective long-term therapy. Despite advances in targeted treatments, patients with brain metastasis continue to exhibit poor prognosis and impaired quality of life^1^. Moreover, the formation of brain metastases as a multistep process is poorly understood. Clarifying the biology of brain metastases is essential for both the prediction of patients at risk to develop brain metastases and the discovery of novel therapeutic targets.

A central question in the brain metastasis field relates to the extent of breakdown of the blood–brain barrier (BBB), the protective lining of blood vessels in the brain, to form a blood–tumor barrier. Distant metastasis formation comprises a multistep process that is often referred to as the metastatic cascade. Disruption of the BBB and a change in the composition of the extracellular matrix by brain tumors can render the BBB leaky at the tumor site^2^. Bos *et al*.^3^ reported that gene expression analysis of brain metastatic cells and clinical samples identified the genes for cyclooxygenase (*COX2*, also known as prostaglandin-endoperoxide synthase 2: *PTGS2*), the epidermal growth factor receptor (EGFR) ligand heparin binding EGF like growth factor (*HBEGF*), and an *a*2,6-sialyltransferase (*ST6GALNAC5*) as mediators of cancer cell passage through the BBB^3^. Moreover, based on information from The Cancer Genome Atlas database, genes encoding GALNT9, an initiator of O-glycosylation, CCDC8, a regulator of microtubule dynamics, and BNC1, a transcription factor with a broad range of targets, have been reported to play a role in the progression of primary breast tumors to brain metastases^4^. In turn, Silva *et al*.^5^ and Vareslija *et al*.^6^ analyzed 39 and 21 matched pairs of primary breast cancers and brain metastases, revealing that the genes for human epidermal growth factor receptor 3 (ERBB3) and RET were significantly overexpressed in brain metastases relative to matched primary tumors.

Another key question to brain metastases is the role of immune modulation. The intact brain contains almost no lymphocytes; however, T and B cells have been observed in the milieus of brain metastases^7^. The overall percentage of tumor-infiltrating lymphocytes (TILs) out of all live cells was shown to be significantly higher in primary breast cancers than that in metastatic tumor samples, which did not change according to each breast cancer subtype^8^. Moreover, brain metastases contained fewer TILs relative to metastatic breast cancers from other sites^9^. More recently, we also reported fewer TILs in brain metastases than in primary breast cancers by paired matched samples^10^.

In addition, several clinical trials to test the efficacies of checkpoint inhibitors against brain metastases are ongoing. The epithelial-mesenchymal transition (EMT) also constitutes a key developmental program that is often activated during cancer invasion and metastasis^11^. Several key inducers of EMT are associated with the ability of breast cancer cells to enter the circulation and seed metastases^12^. However, these studies to elucidate the biological process of brain metastases relied mainly upon *in vitro*/*in vivo* observations as it is challenging to access brain samples. Thus, few validations have been reported using matched pairs of human primary breast cancers and brain metastases.

In this study, we therefore performed gene expression analyses on 16 paired matched samples between primary breast cancers and brain metastases, all of which were collected in the course of clinical care. Our objectives were to determine whether (i) clinic-pathological markers and immune-related gene signatures differed between primary breast cancers and brain metastases; (ii) previously reported genes significantly associated with brain metastases and EMT were reproducible and consistent in our dataset; and (iii) novel therapeutic targets for brain metastases could be identified among agents that have been already approved by the U.S. Food & Drug Administration (FDA) or investigated in clinical trials as molecular target agents for distinct cancers.

## Results

We isolated enough RNA from the 16 patients with paired matched samples. Patient characteristics were shown in Table 1. Of sixteen paired patients, two had brain metastases when first diagnosed with breast cancer; the remaining were diagnosed with brain metastases subsequent to treatment for early or advanced breast cancer. Average age at diagnosis with brain metastases was 56.5 years (min 43.9, max 70.6). Among the 16 patients, six were hormone receptor (Estrogen receptor [ER] and/or Progesterone receptor [PgR]) positive and seven were human epidermal growth factor receptor 2 (HER2) positive as assessed immunohistochemistry in Primary breast cancers. Two received stereotactic irradiations prior to brain surgery.

**Table 1:** Patients characteristics*.

Age		
Average (range.)	56.5 (43.9–70.6)	
	Number of patients	%
**ER**		
positive	6	37.5%
negative	9	56.3%
Unknown	1	6.3%
PgR		
positive	4	25.0%
negative	11	43.8%
Unknown	1	6.3%
HER2		
positive	7	43.8%
negative	7	43.8%
Unknown	2	12.5%
de novo Stage IV or Rec.		
de novo Stage IV	2	12.5%
Rec.	14	82.4%
Systemic chemotherapy		
Yes	14	87.5%
No	2	12.5%
Radiation therapy		
Yes	2	12.5%
No	14	87.5%

*Age: Age at brain surgery; ER: Estrogen receptor; PgR: Progesteron receptor; HER2: Humane epidermale groeifactor receptor 2; Rec.: Recurrence; Systemic chemotherapy: Systemic chemotherapy before brain surgery; Radiation therapy before brain surgery.

**Figure 1:**
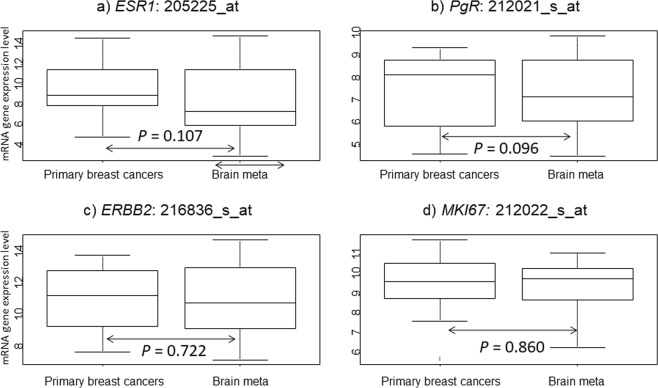
mRNA gene expression in the two groups (Primary breast cancers and Brain meta). (**a**) *ESR1*: 205225_at, (**b**) *PgR*: 212021_s_at, (**c**) *ERBB2*: 216836_s_at, and (**d**) *MKI67:* 212022_s_at. *P* values were calculated using the paired samples Wilcoxon test.

We first performed paired class comparison tests in two groups (Primary breast cancers/Brain meta) for classical clinical markers (Fig. 1). No significant differences were noted in all four genes (*ESR1*, *PgR*, *ERBB2* and *MKI67*) mRNA expression between Primary breast cancers and Brain meta groups.

**Figure 2:**
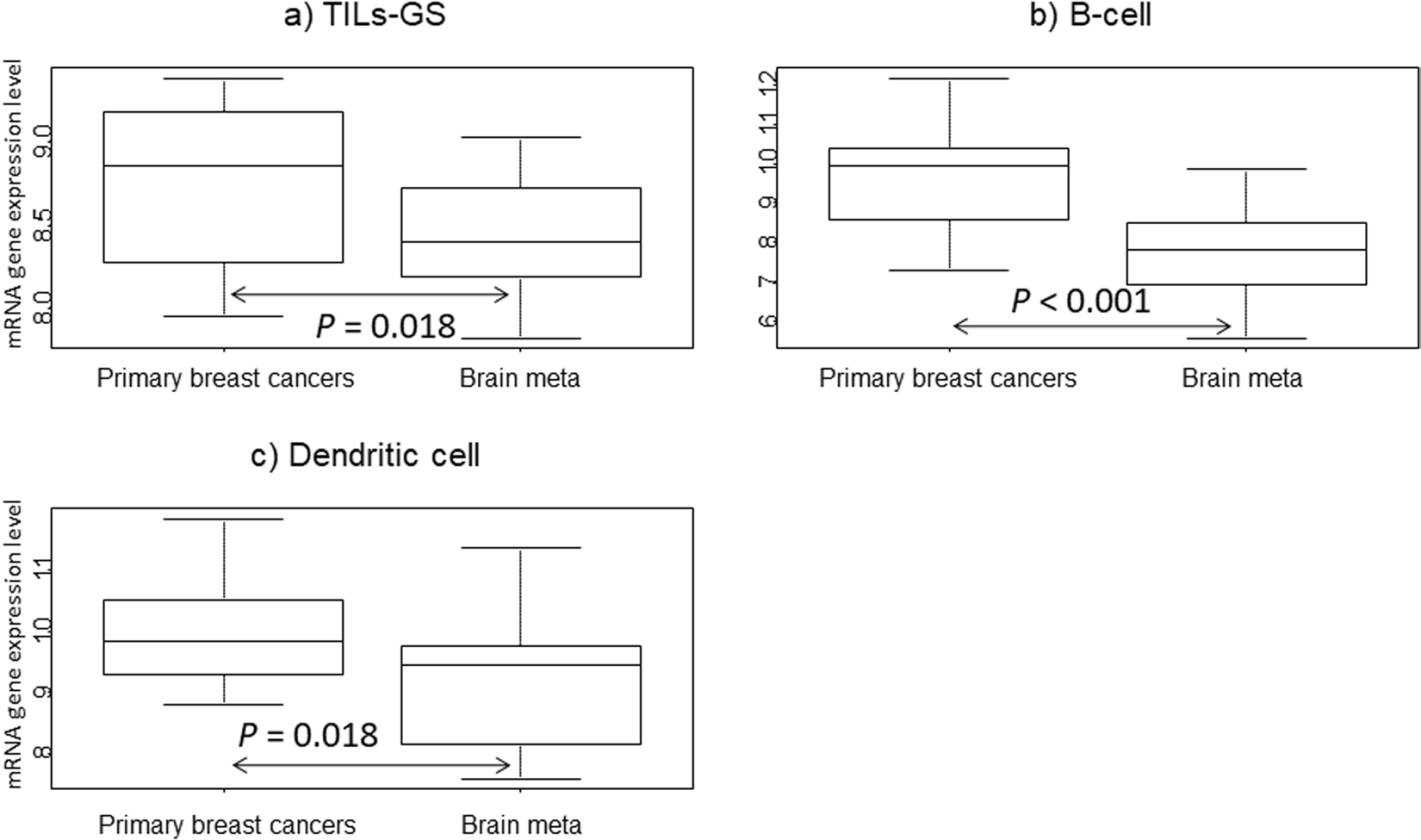
mRNA gene expression in the three groups (Primary breast cancers and Brain meta) for immune-related gene signatures. (**a**) TILs-GS^32^, (**b**) B-cell^31^, and (**c**) Dendritic cell^31^. *P* values were calculated using the paired samples Wilcoxon test.

Similar paired analyses were also performed for immune-related signatures, which were calculated based on average gene expression (Fig. 2). The list of genes is shown in Supplementary Table 1.In the Brain meta group, all three signatures (TILs-GS: *P* = 0.018, B-cell: *P* < 0.001 and Dendritic cell: *P* = 0.018) gene expression was significantly decreased compared to that in Primary breast cancers. Similar paired class comparison tests in the two groups for seven genes associated with brain metastases identified from previous reports were also performed (Table 2). No genes were identified as being differentially expressed between Primary breast cancers and Brain meta group.

**Table 2:** Class comparison test for genes associated with brain metastases in tumor groups.

Symbol	Probe set	*P* value	Geometric mean of intensities (Primary breast cancers /Brain meta)
*BNC1*	206581_at	0.98	0.99
*ST6GALNAC5*	220979_s_at	0.95	0.97
*PTGS2*	204748_at	0.55	0.72
*HBEGF*	38037_at	0.23	1.46
*RET*	205879_x_at	0.28	1.38
*EGFR*	201984_s_at	0.58	1.27
*ERBB3*	202454_s_at	0.89	1.04

Next, paired similar class comparison test with EMT and the two groups (Primary breast cancers/Brain meta) was performed to determine whether events of EMT occurred in brain metastases (Table 3). Only one (*CDH2*) of the 13 EMT-related genes exhibited significantly higher gene expression in the Brain meta compared to the Primary breast cancers group (*P* < 0.001). Two (*FN1*, and *VIM*) of the 13 genes presented significantly higher gene expression in the Primary breast cancers than Brain meta group, although those two genes have been reported over-expression in metastatic sites^11–13^.

**Table 3:** Class comparison test for EMT related genes in tumor groups.

Symbol	Probe set	*P* value	Geometric mean of intensities (Primary breast cancers/Brain meta)
*CDH2*	203440_at	0.02	0.30
*CTNNA1*	200765_x_at	0.31	0.82
*JUP*	201015_s_at	0.51	0.86
*CDH1*	201131_s_at	0.60	0.86
*GEMIN2*	205063_at	0.71	0.86
*TCF3*	213732_at	0.79	0.87
*FN1*	214702_at	0.01	2.57
*VIM*	201426_s_at	0.03	1.92
*SNAI2*	213139_at	0.06	2.00
*TWIST1*	213943_at	0.12	1.77
*ZEB2*	203603_s_at	0.68	1.15
*SNAI1*	219480_at	0.75	1.18
*FOXC2*	214520_at	0.83	1.07

**Figure 3:**
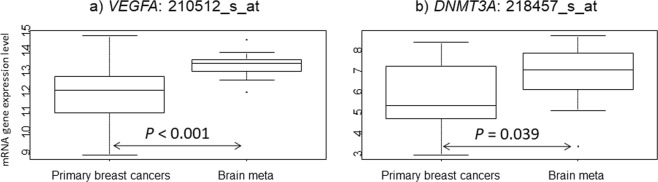
mRNA gene expression in the three groups (Primary breast cancers, and Brain meta). (**a**) *VEGFA*: 210512_s_at and (**b**) *DNMT3A*: 218457_s_at. *P* values were calculated using the paired samples Wilcoxon test.

Finally, we tested 41 genes to identify potential new therapeutic targeted genes for brain metastases and performed a class comparison test using the paired samples Wilcoxon test (Supplementary Table 2). Only two targets (*VEGF-A*: *P* < 0.001 and *DNMT3A*: *P* = 0.039) among the 41 genes exhibited significantly higher gene expression in the Brain meta than Primary breast cancers group (Fig. 3).

## Discussion

To our knowledge, this is the first study to systemically examine gene expression differences between pair-matched primary breast cancers and brain metastases. We have collected a unique set of clinical material through collaborations with multiple institutions and involving brain metastases that are rarely excised. The analysis of this resource has tried to reveal the mechanisms of breast cancer colonization of the brain along with novel potential therapeutic agents. The present study showed that immune-related gene signatures exhibited significantly lower mRNA expression in brain metastases than that in primary breast cancers and early breast cancers without metastases. Micro-environments in brain metastases, a so-called “immune desert”, are consistent with the findings of our previous reports^10,14^ and may lead to the observed resistance.

Recently, immune checkpoint inhibitors have been reported to show modest efficacy in breast cancers. For example, atezolizumab plus nab-paclitaxel prolonged progression-free survival among patients with metastatic triple-negative breast cancer^15^. In addition, several clinical trials (NCT03449238, NCT03483012, NCT03417544, NCT02563925, and NCT02563925) to test the efficacy of immune checkpoint inhibitors for patients with breast cancer patients and brain metastases are ongoing (https://clinicaltrials.gov/ accessed January 10, 2019). Notably, all of these ongoing trials assessed the combinations of immune checkpoint inhibitors with other therapies including radiation, chemotherapies, or targeted therapies, rather than a single targeted drug. The effect of single agent may be limited from our results. A clinical trial (NCT02669914) to test the effects of a single immune checkpoint (PD-L1) inhibitor in patients with brain metastases was terminated because of low accrual and funding has been withdrawn.

We also identified the candidate therapeutic target genes for patients with brain metastases, *VEGFA* and *DNMT3A*, by analyzing 41 FDA-approved or under-investigation agents. VEGF-A serves as a primary factor driving expansion of the tumor vascular bed. In many instances, vascular control has been reported to lead to decreased tumor growth, despite the fact that the normalized tumor vessels appear more functional^16^. Bevacizumab constitutes a humanized monoclonal antibody directed against all isoforms of VEGF-A. The addition of bevacizumab to chemotherapy for patients with metastatic breast cancers produced significant clinical efficacy in phase III trials^17,18^. VEGF also plays a significant role in BBB breakdown and regulates focal adhesion assembly in human brain microvascular endothelial cells through activation of the focal adhesion kinase^19^. Several factors have been described to play a key role in this process. For example, VEGF may contribute to brain metastases formation by enhancing the trans endothelial migration of tumor cells through the downregulation of endothelial integrity^20^. In preclinical analyses, a significant increase in *VEGFA* production was observed in a brain metastases breast cancer cell line (MDA-MB-231-BR) compared with that in the parental cell line (MDA-MB-231), which corresponds to brain metastases lesions with significantly more CD31-positive blood vessels following intra-carotid injection of breast cancer cells in mice^21^. In another recent preclinical mouse model, bevacizumab with chemotherapy resulted in antitumor activity in a metastatic setting^22^. Improved progression-free survival and maintenance of baseline quality of life and performance status for patients with newly diagnosed glioblastoma were also observed with bevacizumab^23^. Moreover, for breast cancers with brain metastases, several phase II trials (NCT01004172, NCT01281696, and NCT00476827) have been completed (https://clinicaltrials.gov/ accessed January 10, 2019). However, no final reports have been published and no phase III trials have been initiated to date.

In turn, *DNMT3A*, along with *DNMT3B*, functions as a *de novo* methyltransferase that plays important roles in normal development especially during early embryogenesis, as well as in embryonic carcinoma cells^24^. Deregulation of *DNMT3A* and *DNMT3B* is associated with various human diseases including hematological cancer^25^. DNA methyltransferase inhibitors have been recognized as promising candidate anticancer drugs and are widely used to treat patients with acute myeloid leukemia and myelodysplastic syndromes^26,27^. In a phase III trial for myelodysplastic syndromes, treatment with azacitidine, a DNMT inhibitor, improved overall survival compared to conventional care^28^. Furthermore, 5-azacytidine, a global DNA methyltransferase inhibitor, was approved to treat myelodysplastic syndromes by the FDA and is being clinically tested for solid tumors including brain tumor (NCT03206021, NCT02940483, and NCT03572530). An ongoing Phase I clinical trial (NCT02223052) is also assessing the efficacy of azacitidine for various hematological and solid cancers including metastatic breast cancer.

Among 19 genes (13 related to EMT and 6 related to brain metastasis), we compared between primary and brain metastasis sites. Only one gene, *CDH2*, also known as N-cadherin (Neueonal), exhibited significant differences in the two groups. N-cadherin gene expression was upregulated, paralleled by the reduced expression of E-cadherin^29^. Notably, the inhibition of N-cadherin gene expression had previously been tested as a strategy to reduce the proliferation and invasion of cancer cells *in vitro*^29^. The remaining genes had no significant differences between primary and brain metastases. These are in contrast to several published reports that found significant gene expression differences in two groups^3–5,11–13^. These discrepancies may be related to differences in human samples and preclinical analyses. For example, the majority of published studies were based on preclinical analyses, whereas only a single report derived from paired samples between primary breast cancers and brain metastases;^5^ however, this previous analysis utilized a different platform for determining gene expression than that in the present study. Moreover, some of the previous reports used reverse transcription polymerase chain reaction analysis to quantify gene expression differences, which exhibits a different sensitivity and dynamic range of mRNA detection compared with those of gene expression arrays.

This study has several limitations. First, the sample size was small. Samples from paired primary tumors and brain metastases are quite rare because the number of surgeries performed for brain metastases has decreased owing to advances in radiotherapy treatments. Furthermore, it is difficult to isolate RNA with high quality from old FFPE sample. The low number of identified significant genes by class comparison test in our analyses might derive from the small sample size and may represent false positives. Moreover, the present study relied on retrospective data collected from multiple institutions. Different institutions may have different ways to collect and fix samples. In addition, two samples from brain metastases were collected following radiation therapy. Fourteen patients got brain metastases after various systemic treatments for advanced breast cancers and/or the other metastatic sites. These differences in baseline conditions may contribute to the variation of results. Despite these limitations, our observations indicate that brain metastases exhibited suppressed immune-related functions compared to those of primary breast cancers, which is consistent with previously reported data.

In conclusions we revealed that there are distinct gene expression profiles in early primary breast cancers, primary breast cancers with brain metastases, and brain metastases. These results may facilitate the development of novel therapeutic strategies for brain or other metastases in consideration of gene expression characteristics. Further studies are needed to explore whether these distinct expression patterns are associated with the causes or consequences of the cancer status, and to compare gene expression profiles between metastases to the brain and other common sites (e.g., the lung, bone, and liver).

## Methods

### Patient samples and gene expression profiling

**Figure 4:**
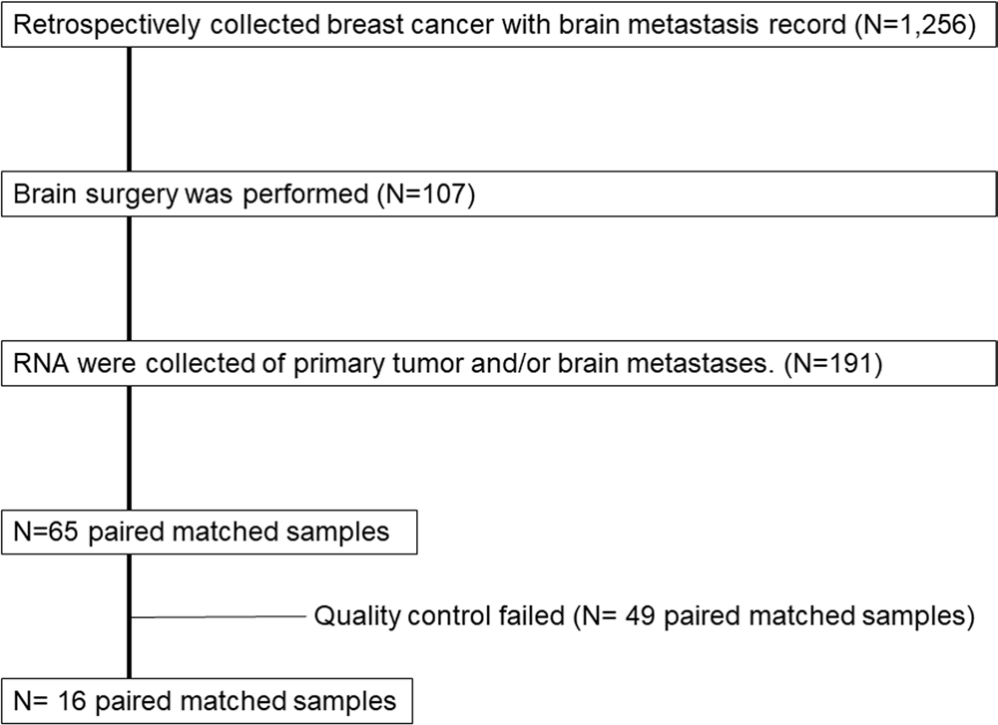
Workflow.

This investigation was based on a previous study performed by the Japan Clinical Oncology Group (JCOG) Breast Cancer Study Group. The eligibility criteria for the original study have been described previously^30^. A workflow was depicted in Fig. 4. We collected data for 1,256 patients with brain metastases from breast cancer between April 1, 2001 and December 31, 2012 was compiled from 8 institutions. Among them, 107 patients with breast cancer who were diagnosed with brain metastases and who underwent surgery. We received 191 samples that included pair-matched samples of both the primary tumor and brain metastasis as well as brain metastasis samples only. We collected RNA from the all formalin-fixed paraffin-embedded (FFPE) sample, however most of sample could not collect enough RNA to analyze gene expression. A total of 16 paired matched samples from primary breast cancers and brain metastases diagnosed were collected from eight institutions. Brain metastases were identified based on magnetic resonance imaging and/or computed tomography findings. The clinical characteristics of all the patients were obtained from their medical records. This retrospective study was approved by the institutional review board of each participating institute (Tokai University School of Medicine; National Hospital Organization Osaka National Hospital; Kinki University School of Medicine; Niigata Cancer Center Hospital; Shizuoka General Hospital; Hokkaido Cancer Center; National Hospital Organization, Tokyo Medical Center; and Gunma Prefectural Cancer Center). All the review boards which approved the study waived the need for informed consent. All experiments were performed in accordance with relevant guidelines and regulations.

Matched FFPE primary breast cancer and brain metastasis specimens for gene expression analysis were collected. RNA from the specimens was isolated, and quantity and quality of each RNA was ascertained using an Agilent 2100 Bioanalyzer (Agilent Technologies, United States, CA). Genome-wide expression levels of transcripts were analyzed using Affymetrix U133A gene chips according to the manufacturer’s instructions. Complete gene expression data are available in the Gene Expression Omnibus (GEO: https://www.ncbi.nlm.nih.gov/gds) under accession number GSE125989. All gene expression data were normalized using the MAS5 algorithm (http://www.bioconductor.org) with the mean expression centered to 600 and log 2 transformed. If a given gene was represented by two or more probe sets, we retained only a single probe set with the highest average gene expression. We defined HER2 positivity as having an immunohistochemistry score of 3+ or a positive fluorescence *in situ* hybridization result. Hormone receptor (ER/PgR) positivity was diagnosed if at least 1% of nuclei in the tumor were stained on immunohistochemical tests for ER/PgR.

### Statistical analyses

We defined two groups; samples of primary breast cancers with brain metastases: “Primary breast cancers” and samples of brain metastases: “Brain meta” and compared gene expression level between each group using Paired Samples Wilcoxon test. First, we compared classical clinical pathological markers (*ESR1*: 205225_at, *PgR*: 212021_s_at, *ERBB2*: 216836_s_at, and *MKI67:* 212022_s_at) in paired two groups. Second, immune-related signatures were compared in the two groups in order to assess the immune microenvironments in brain metastases. Immune-related signatures (TILs-GS, B-cell, and Dendritic cell) have been previously reported^31,32^. Next, we performed pared class comparison test for EMT-related genes (*CDH1, CDH2, CTNNA1, FN1, FOXC2, GEMIN2, JUP, SNAI1, SNAI2, TCF3, TWIST1, VIM*, and *ZEB2*) selected according to previous studies^11–13^, retaining only genes annotated in the Affymetrix U133A gene chips. Of thirteen EMT-related genes, three (*CDH1*, *CTNNA1*, and *JUP*) were under-expressed and the remaining ten (*TWIST1*, *TCF3, SNAI1*, *SNAI2*, *GEMIN2*, *CDH2*, *VIM*, *FN1*, *FOXC2*, and *ZEB2)* were over-expressed by the induction of EMT as previously reported papers^11–13^.

Next, we selected seven genes (*EGFR*, *PTGS2*, ST6 N-acetylgalactosaminide alpha-2,6-sialyltransferase 5 [*ST6GALNAC5*], *HBEGF*, epidermal growth factor receptor 3 [*ERBB3*], basonuclin 1 [*BNC1*] and *RET*) associated with brain metastases from previous reports^3–6^. Class comparison tests for the seven genes were performed in the paired two groups (Primary breast cancers/Brain meta).

Finally, we selected 41 genes that are targeted by FDA-approved drugs or have been investigated in clinical trials as molecular target agents for distinct cancers, including breast cancer, to explore new therapeutic targets for patients with breast cancer exhibiting brain metastases. The information on anticancer therapy drugs was obtained from the National Cancer Institute drug information^33^, Drug@FDA^34^, and Clinical Trials.gov^35,36^. Several of the 41 selected genes were associated with DNA damage repair pathways and BRCA functions (*BRCA1*, *BRCA2*, *PARP1*, and *PARP2*), cyclin dependent kinase (CDK) pathways (*CDK2*, *CDK4*, *CDK6*, *CCND1*, *CDKN2A*, and *RB1*), vascular endothelial growth factor (VEGF) and VEGF receptor pathways (*VEGF-A*, *VEGF-B*, *VEGF-C*, *EGFR*, *PGF*, *KDR*, and *FTL4*), modulation of DNA methylation and histone acetylation (*HDAC1*, *HDAC2*, *HDAC3*, DNA methyltransferase 1 (*DNMT1*), *DNMT3A*, and *DNMT3B*), immune responses (*PDCD1LG2*), and the mTOR pathway (*mTOR* and *PIK3CA*). Others (*AR*, *ERBB3* and *p53, AKT1*, *ALK*, *RAF1*, *CTNNB1*, *MET*, *STK11*, *PTEN*, *NF1*, *ROS1*, *NOTCH1*, *ATM*, *KITL*, and *KRAS*) were also FDA-approved drugs or under investigation for cancers including breast cancer. We performed class comparison testing in the two groups.

All statistical analyses were performed using BRB-ArrayTools version 3.9.0a (http://linus.nci.nih.gov/BRB-ArrayTools.html) and R software version 2.7.2 (http://www.r-project.org). Two-sided P values ≤ 0.05 were considered statistically significant.

### Ethics approval and consent to participate

This retrospective study was approved by the institutional review board and a waiver of consent has been obtained from a research ethics committee. All the review boards which approved the study waived the need for informed consent

## Supplementary information

Supplementary Info

## Data Availability

Complete gene expression data are available in the Gene Expression Omnibus (GEO: https://www.ncbi.nlm.nih.gov/gds) under accession number GSE125989.
