# Supplementary material for: Distinct gene expression profiles between primary breast cancers and brain metastases from pair-matched samples
Source: Sci Rep. 2019 Sep 16;9:13343. doi: 10.1038/s41598-019-50099-y (PMC6746866; doi:10.1038/s41598-019-50099-y)
Supplement: Supplementary file 1 — Supplementary Info [file 41598_2019_50099_MOESM1_ESM.pdf]

Research article

Distinct gene expression profiles between primary breast cancers and brain metastases from pair-matched samples

Takayuki Iwamoto[1], Naoki Niikura[2*], Rin Ogiya[2], Hiroyuki Yasojima[3], Ken-ichi Watanabe[4], Chizuko Kanbayashi[5], Michiko Tsuneizumi[6], Akira Matsui[7], Tomomi Fujisawa[8], Tsutomu Iwasa[9], Tadahiko Shien[1], Shigehira Saji[10], Norikazu Masuda[3] and Hiroji Iwata[11]

Sup.Table 1-A List of genes for TILs-GS

Probe set	Symbol
200629_at	*WARS*
201762_s_at	*MIR7703*
201930_at	*MCM6*
203416_at	*CD53*
204170_s_at	*CKS2*
204563_at	*SELL*
204822_at	*TTK*
204891_s_at	*LCK*
205255_x_at	*TCF7*
205569_at	*LAMP3*
205671_s_at	*HLA-DOB*
205798_at	*IL7R*
207551_s_at	*MSL3*
208687_x_at	*SNORD14C*

208778_s_at	*SNORA29*
208864_s_at	*TXN*
208885_at	*LCP1*
210029_at	*IDO1*
210439_at	*ICOS*
210821_x_at	*CENPA*
212314_at	*SEL1L3*
212718_at	*PAPOLA*

Sup.Table 1-B List of genes for B-cell

Probe set	Symbol
201209_at	*HDAC1*
201244_s_at	*RAF1*
201533_at	*CTNNB1*
201697_s_at	*DNMT1*
201746_at	*TP53*
201833_at	*HDAC2*
201984_s_at	*EGFR*
202246_s_at	*CDK4*
202454_s_at	*ERBB3*
203132_at	*RB1*
203683_s_at	*VEGFB*
203934_at	*KDR*
204053_x_at	*PTEN*
204252_at	*CDK2*
204369_at	*PIK3CA*
204752_x_at	*PARP2*
206846_s_at	*HDAC6*
207029_at	*KITLG*
207163_s_at	*AKT1*
207569_at	*ROS1*
208211_s_at	*ALK*
208644_at	*PARP1*

208712_at	*CCND1*
209138_x_at	*IGLC1*
209374_s_at	*IGHM*
209644_x_at	*CDKN2A*
209652_s_at	*PGF*
209946_at	*VEGFC*
210316_at	*FLT4*
210512_s_at	*VEGFA*
210858_x_at	*ATM*
211110_s_at	*AR*
211430_s_at	*IGHG2*
211599_x_at	*MET*
211634_x_at	*IGHM*
211635_x_at	*IGHG4*
211637_x_at	*IGHG4*
211641_x_at	*IGHV4-31*
211643_x_at	*IGK*
211644_x_at	*IGK*
211645_x_at	*IGKC*
211798_x_at	*IGLJ3*
211851_x_at	*BRCA1*
212592_at	*JCHAIN*
212676_at	*NF1*
214160_at	*CDK6*
214352_s_at	*KRAS*
214669_x_at	*IGKC*
214677_x_at	*IGLC1*
214727_at	*BRCA2*
214768_x_at	*IGKC*
214777_at	*IGKC*
214836_x_at	*IGK*
214916_x_at	*IGHV3-23*
214973_x_at	*IGHD*
215121_x_at	*CYAT1*
215176_x_at	*IGK*
215379_x_at	*IGLV1-44*

215946_x_at	*IGLL3P*
216207_x_at	*IGKC*
216401_x_at	*LOC642424*
216557_x_at	*IGHV4-31*
216576_x_at	*IGK*
216984_x_at	***IGLV@***
217022_s_at	*IGHA2*
217148_x_at	*IGLV@*
217157_x_at	*IGK*
217179_x_at	*IGLV1-51*
217227_x_at	*IGLV1-44*
217235_x_at	*IGLL5*
217378_x_at	*LOC100130100*
217480_x_at	*LOC642838*
218457_s_at	*DNMT3A*
218902_at	*NOTCH1*
220049_s_at	*PDCD1LG2*
220668_s_at	*DNMT3B*
221651_x_at	*IGK*
221671_x_at	*IGK*
41657_at	*STK11*

Sup.Table 1-C List of genes for Dendritic cell

Probe set	Symbol
203473_at	*SLCO2B1*
208894_at	*HLA-DRA*
209619_at	*CD74*
209823_x_at	*HLA-DQB1*
210982_s_at	*HLA-DRA*
211990_at	*HLA-DPA1*
211991_s_at	*HLA-DPA1*
212671_s_at	*HLA-DQA2*
212998_x_at	*HLA-DQB1*

215193_x_at	*HLA-DRB4*
218232_at	*C1QA*
220532_s_at	*TMEM176B*

Sup.Table 1-D List of genes for EMT

Probe set	Symbol
200765_x_at	*CTNNA1*
201015_s_at	*JUP*
201131_s_at	*CDH1*
201426_s_at	*VIM*
203440_at	*CDH2*
203603_s_at	*ZEB2*
205063_at	*GEMIN2*
213139_at	*SNAI2*
213732_at	*TCF3*
213943_at	*TWIST1*
214520_at	*FOXC2*
214702_at	*FN1*
219480_at	*SNAI1*

Sup. Table 2 Class comparison test for targetable genes in tumor groups

Symbol	Probe set	*P* value	Geometric mean of intensities (Primary breast cancer /Brain meta)
VEGFA	210512_s_at	<0.001	0.35
DNMT3A	208644_at	0.039	0.61
PARP1	218457_s_at	0.053	0.47
MET	211599_x_at	0.20	0.66
RAF1	201244_s_at	0.23	0.67
BRCA2	214727_at	0.25	0.56
CDKN2A	209644_x_at	0.30	0.83
PIK3CA	204369_at	0.37	0.72
DNMT1	201697_s_at	0.39	0.76
AR	211110_s_at	0.42	0.64
CCND1	208712_at	0.44	0.82
NF1	212676_at	0.53	0.86
TP53	201746_at	0.54	0.86
ATM	210858_x_at	0.61	0.87
ALK	208211_s_at	0.71	0.88
KRAS	214352_s_at	0.82	0.92
KITLG	207029_at	0.85	0.93
HDAC1	201209_at	0.93	0.95
DNMT3B	220668_s_at	0.95	0.98
AKT1	207163_s_at	0.95	0.98
ROS1	207569_at	0.08	2.42
PDCD1LG2	220049_s_at	0.035	2.22
KDR	203934_at	<0.001	1.78
FLT4	210316_at	0.12	1.51
CDK2	204252_at	0.28	1.45
PARP2	204752_x_at	0.35	1.44
BRCA1	211851_x_at	0.46	1.42
VEGFB	203683_s_at	0.36	1.4
CDK4	202246_s_at	0.11	1.37
VEGFC	209946_at	0.45	1.33
CDK6	214160_at	0.23	1.31
EGFR	201984_s_at	0.58	1.27

PTEN	204053_x_at	0.45	1.16
CTNNB1	201533_at	0.60	1.14
PGF	209652_s_at	0.72	1.13
NOTCH1	218902_at	0.73	1.13
RB1	203132_at	0.79	1.11
STK11	41657_at	0.84	1.06
HDAC6	206846_s_at	0.82	1.04
ERBB3	202454_s_at	0.89	1.04
HDAC2	201833_at	0.99	1.00
